# *In Vitro* and *In Vivo* Miltefosine Susceptibility of a *Leishmania amazonensis* Isolate from a Patient with Diffuse Cutaneous Leishmaniasis

**DOI:** 10.1371/journal.pntd.0002999

**Published:** 2014-07-17

**Authors:** Adriano C. Coelho, Cristiana T. Trinconi, Carlos H. N. Costa, Silvia R. B. Uliana

**Affiliations:** 1 Departamento de Parasitologia, Instituto de Ciências Biomédicas, Universidade de São Paulo, São Paulo, São Paulo, Brazil; 2 Departamento de Medicina Comunitária, Universidade Federal do Piauí, Teresina, Piauí, Brazil; University of Antwerp, Belgium

## Abstract

Miltefosine was the first oral compound approved for visceral leishmaniasis chemotherapy, and its efficacy against *Leishmania donovani* has been well documented. *Leishmania amazonensis* is the second most prevalent species causing cutaneous leishmaniasis and the main etiological agent of diffuse cutaneous leishmaniasis in Brazil. Driven by the necessity of finding alternative therapeutic strategies for a chronic diffuse cutaneous leishmaniasis patient, we evaluated the susceptibility to miltefosine of the *Leishmania amazonensis* line isolated from this patient, who had not been previously treated with miltefosine. *In vitro* tests against promastigotes and intracellular amastigotes showed that this parasite isolate was less susceptible to miltefosine than *L. amazonensis* type strains. Due to this difference in susceptibility, we evaluated whether genes previously associated with miltefosine resistance were involved. No mutations were found in the *miltefosine transporter* gene or in the *Ros3* or *pyridoxal kinase* genes. These analyses were conducted in parallel with the characterization of *L. amazonensis* mutant lines selected for miltefosine resistance using a conventional protocol to select resistance *in vitro*, i.e., exposure of promastigotes to increasing drug concentrations. In these mutant lines, a single nucleotide mutation G852E was found in the *miltefosine transporter* gene. *In vivo* studies were also performed to evaluate the correlation between *in vitro* susceptibility and *in vivo* efficacy. Miltefosine was effective in the treatment of BALB/c mice infected with the *L. amazonensis* type strain and with the diffuse cutaneous leishmaniasis isolate. On the other hand, animals infected with the resistant line bearing the mutated *miltefosine transporter* gene were completely refractory to miltefosine chemotherapy. These data highlight the difficulties in establishing correlations between *in vitro* susceptibility determinations and response to chemotherapy *in vivo*. This study contributed to establish that the miltefosine transporter is essential for drug activity in *L. amazonensis* and a potential molecular marker of miltefosine unresponsiveness in leishmaniasis patients.

## Author Summary

*Leishmania amazonensis* is the etiological agent of diffuse cutaneous leishmaniasis. The disease is extremely difficult to treat and frequently relapses once the treatment is interrupted. Although not yet approved in Brazil, miltefosine is an attractive alternative for leishmaniasis treatment due to its oral administration and low incidence of side effects. Here, we evaluated the efficacy of miltefosine against a *L. amazonensis* line that was isolated from a chronic diffuse cutaneous leishmaniasis patient to ascertain whether miltefosine could be considered as a therapeutic option in this case. Parasites isolated from this patient were less susceptible to miltefosine than a reference strain *in vitro*. The mechanisms underlying this decreased susceptibility were studied in this natural parasite isolate in parallel with mutant strains selected *in vitro* for miltefosine resistance. A mutation in the gene encoding the miltefosine transporter was identified in the mutants selected *in vitro* but not in the line isolated from the patient. Notwithstanding the decreased susceptibility *in vitro*, when used to treat infected mice, miltefosine was equally effective against the isolate from the patient and the type strain, but completely ineffective against the resistant line.

## Introduction

*Leishmania* spp. are the etiological agents of a spectrum of diseases collectively known as leishmaniasis, endemic in tropical and subtropical areas of the world [1]. *Leishmania braziliensis* and *Leishmania amazonensis* are the main causative agents of cutaneous leishmaniasis (CL) in Brazil, with more than 20,000 cases in 2010 [2]. *L. amazonensis* is also the etiological agent of diffuse cutaneous leishmaniasis (DCL), a severe pathology associated with defective cell mediated immune responses to the parasite [3].

Since there is no effective vaccine for prevention, control is essentially based on chemotherapy. Meglumine antimoniate (Glucantime, Aventis) is the drug of choice for the treatment of CL in Brazil [2]. Unresponsive cases are treated with pentamidine or amphotericin B in liposomal or conventional forms. There is, however, no effective treatment for DCL. Clinical improvement is noted during and shortly after the courses of treatment, but in general relapses follow the discontinuation of therapy [4].

Miltefosine (MF), an antitumoral oral compound, has recently emerged as an effective drug against visceral leishmaniasis (VL), with cure rates as high as 95% in India [5]–[7]. For CL, reported cure rates vary between 53% and 91% depending on the *Leishmania* species [8].

MF's leishmanicidal mode of action is not completely understood and most information on its properties was obtained from MF-resistant parasites generated *in vitro*. MF resistance in *Leishmania* is mainly due to a drastic reduction in drug accumulation [9], associated with the inactivation of a P-type ATPase, also known as Miltefosine Transporter (MT), responsible for the translocation of phospholipids across the plasma membrane of the parasite [10]. Mutated *MT* were observed in resistant *L. donovani* [10], *L. major* and *L. infantum* [11]. The uptake of MF is also dependent on another protein, LdRos3, a non-catalytic subunit of the MT. LdRos3 inactivation also led to a significant reduction of MF accumulation in *L. donovani* [12]. Pyridoxal kinase (PK), an enzyme involved in the formation of pyridoxal 5′-phosphate (the active form of vitamin B6) also has a role in MF susceptibility [11].

MF susceptibility data for *L. amazonensis* strains is limited, especially in Brazil, where the drug has yet to be approved for leishmaniasis treatment. Miltefosine's EC_50_ has been determined for three *L. amazonensis* type strains [13]–[15], but whether differences in MF susceptibility occur in *L. amazonensis* field isolates is entirely unknown.

In this study, we evaluated the susceptibility to MF of *L. amazonensis* 2506, a strain recently isolated from a chronic DCL patient in Teresina city, Piauí state, Brazil. The patient had previously received multiple courses of antimony and amphotericin B, with only partial responses to chemotherapy but had not been exposed to miltefosine. Known molecular signatures for MF resistance and phospholipid uptake were evaluated in this isolate and in an *in vitro* selected MF resistant *L. amazonensis* mutant.

## Methods

### Ethics statement

Animal experiments were approved by the Ethics Committee for Animal Experimentation (Protocol: 178/138/02) in agreement with the guidelines of the Sociedade Brasileira de Ciência de Animais de Laboratório (SBCAL) and of the Conselho Nacional de Controle da Experimentação Animal (CONCEA).

### Chemical compounds

MF was purchased from Sigma-Aldrich (St. Louis, MO, USA); 2-(6-(7-nitrobenz-2-oxa-1,3-diazol-4-yl)amino)hexanoyl-1-hexadecanoyl-*sn*-glycero-3-phosphocholine and N-(7-nitrobenz-2-oxa-1,3-diazol-4-yl)-1,2-dihexadecanoyl-*sn*-glycero-3-phosphoethanolamine, triethylammonium salt (NBD-PC and NBD-PE respectively) were obtained from Molecular Probes.

### Parasites, culture conditions and selection of MF resistant mutants

The clinical isolate 2506 (MHOM/BR/2008/2506) was obtained in 2008 from a diffuse cutaneous leishmaniasis patient. Parasites were cultured from aspiration of skin lesions performed as part of the follow up procedure and with the patient's consent. Material collected from the border of the skin lesion was suspended in biphasic Schneider's and Novy, McNeal, and Nicolle media and incubated at 26°C. Promastigotes identified in the primary culture were subsequently subpassaged in Schneider's medium and aliquots were frozen. This isolate, named MHOM/BR/2008/2506, was typed by PCR of the internal transcribed ribosomal DNA as described [16] and identified as *L. amazonensis* (data not shown).

*L. amazonensis* reference strain (MHOM/BR/1973/M2269) [17] and the clinical isolate (MHOM/BR/2008/2506) were grown at 25°C in M199 medium (Sigma-Aldrich, St. Louis, MO, USA) supplemented with 10% heat inactivated fetal calf serum (FCS) and 0,25% of hemin. *L. amazonensis* independent mutants were selected from the reference strain M2269 by exposing promastigotes to increasing MF concentrations. Four independent mutants were recovered in the presence of 100 µM MF and cultured in 150 µM of MF, allowing the selection of the MF150.3 line. To obtain clonal lines of mutants, the MF150.3 resistant population was plated onto M199 medium containing 1% of agar (Gibco, Invitrogen Corporation). After 15 days, colonies were picked and expanded in liquid M199 medium containing 150 µM of MF. The stability of mutants was tested by trying to get revertants. This was done by culturing the MF150.3 resistant cloned lines in the absence of MF for 20 passages.

### DNA manipulation

Total genomic DNA was isolated using DNAzol reagent (Invitrogen) as recommended by the manufacturer. Genes involved in MF susceptibility/resistance in *Leishmania* spp. were amplified by PCR using Phusion High-Fidelity DNA Polymerase (New England Biolabs Inc.) or Accuprime Taq DNA polymerase system (Life Technologies). Primers were designed based on the *L. mexicana* (MHOM/GT/2001/U1103) genome data [18] available at TriTrypDB (http://www.tritrypdb.org/). Internal primers were also designed for nucleotide sequencing of studied genes. All primers were designed using Primer3 software [19] and are listed in Table S1. PCR amplified products were excised from 0.8% agarose gels after electrophoresis and purified with QIAquick PCR purification kit (Qiagen). Nucleotide sequences were determined automatically with the Big Dye Terminator v3.1 Cycle Sequencing kit (Applied Biosystems). Nucleotide sequence analysis was performed using Lasergene Software (DNASTAR) and Clone Manager 9.0 Software. Nucleotide sequences are available at GenBank under accession number: KF993340, KF993341 and KF993342. While writing this paper, the genome sequence of the *L. amazonensis* M2269 strain was published [20] and the sequence is now available at http://bioinfo08.ibi.unicamp.br/leishmania/.

### Analysis of the uptake of fluorescent phospholipids

Log-phase promastigotes parasites were incubated in HPMI buffer (5×10^6^/mL) (20 mM HEPES, 132 mM NaCl, 3.5 mM KCl, 0.5 mM MgCl_2_, 5 mM glucose, 1 mM CaCl_2_, pH 7.4) containing 0.3% (w/v) BSA and 10 µM of NBD-PC or NBD-PE for 30 minutes at 25°C [21]. Before the addition of NBD-phospholipids, parasites were incubated for 15 minutes in buffer containing 500 µM PMSF to inhibit the catabolism of phospholipids. After labelling with NBD, parasites were washed twice with ice-cold HPMI containing 0.3% BSA to remove short-chain NBD-phospholipids from the external plasma membrane [22], [23] and then finally resuspended in PBS for flow cytometry analysis.

Labeled parasites were analysed at room temperature using Guava EasyCyte Mini Flow Cytometer System (Millipore). Data from 5,000 cells, defined by gating at data acquisition, was collected and analysed using CytoSoft version 4.2.1 software (Guava Technologies) and FlowJo version 9.4.9 software (Tree Star, Ashland, Oregon).

Alternatively, 1×10^6^ labeled parasites (5×10^6^/mL in PBS) at room temperature were analysed in a microplate reader (POLARstar Omega, BMG Labtech) with excitation at 460 nm and emission at 530 nm. At least three independent experiments were done in duplicate.

### MF activity against promastigotes and intracellular amastigotes

Drug activity was determined by incubating promastigotes in the presence of increasing MF concentrations (3–400 µM). After 24 h of incubation, the number of viable cells was determined as described [24]. Briefly, cells were incubated with MTT (3-[4,5-dimethyl-2-thiazolyl]-2,5- diphenyl-2H-tetrazolium bromide; Sigma-Aldrich) and the optical density was determined in a plate reader (POLARstar Omega, BMG Labtech) with a reference wavelength of 690 nm and a test wavelength of 595 nm. Results were expressed as the mean percentage reduction of parasite numbers compared with untreated control wells calculated for at least three independent experiments performed in triplicate. The EC_50_ was determined by sigmoidal regression curves using Graph Pad Prism 6.0 software.

The activity of MF against intracellular amastigotes was analysed using infected bone marrow-derived macrophages (BMDM) from BALB/c mice as previously described [25]. BMDM were plated on round glass coverslips in 24-well culture dishes, at a density of 4×10^5^ cells in 500 µL of RPMI 1640 medium (Gibco, Invitrogen Corporation) supplemented with 10% FCS (Gibco, Invitrogen Corporation) in a 5% CO_2_ atmosphere for 24 h at 37°C allowing macrophages to adhere. Macrophages were then infected with stationary-phase promastigotes (20 parasites per macrophage) for 3 h at 33°C. Non-internalized parasites were removed by washing with warmed PBS, followed by the addition of fresh medium containing increasing MF concentrations (2.5–40 µM). After 72 h, the cells were fixed in methanol and stained with the Instant Prov kit (Newprov, Pinhais, PR, Brazil). The percentage of infected macrophages was determined by counting 100 cells in three independent experiments.

### MF treatment in mice infected with *L. amazonensis* strains and MF150.3 mutant

Female BALB/c mice were obtained from Instituto de Ciências Biomédicas, Universidade de São Paulo. Considering that the reference, isolate and MF150.3-1 mutant parasites were in different passages in culture *in vitro* as promastigotes and that the number of passages can affect the infectivity of the parasites [26], we normalized the inoculum for BALB/c infections using amastigotes. These were obtained from *in vitro* infections using 1×10^7^ BMDM plated in 148 cm^2^ dishes and infected with stationary phase promastigotes. After 72 h, infected BMDM were detached using a cell scraper and amastigotes were counted with a Neubauer hemocytometer. Amastigotes (1×10^6^) of *L. amazonensis* M2269, 2506 and mutant MF150.3 were injected subcutaneously in the right hind footpad in a volume of 30 µL.

MF treatment was initiated 5 weeks after infection in experimental groups of 5 animals. An untreated group was used as a control for each line studied. MF was prepared daily with sterile water and the treatment was administered in doses of 13 mg/kg/day by oral gavage for 15 consecutive days. Lesion size was evaluated once a week using a caliper (Mitutoyo Corporation, Kawasaki, Kanagawa, Japan) by measuring the difference in the thickness between the infected and contralateral uninfected footpad. Parasite burden in three infected animals from each group was determined by limiting dilution as described [27], one week after the end of treatment. Animals with absence of detectable parasites by limiting dilution and histopathological examination of infected tissues at the end of the treatment were considered cured. MF150.3-1 parasites were recovered from mice after the end of treatment, differentiated into promastigotes *in vitro* in M199 medium and used in MF susceptibility assays during the first passages *in vitro* using MTT as described above.

### Statistical analysis

Statistical analysis was performed using GraphPad Prism 6.0 software. For data on *in vitro* intracellular amastigotes, lesion progression and limiting dilution, we used One Way ANOVA, followed by the Tukey post-test. A result was considered significant at P<0.05.

## Results

### Susceptibility of the *L. amazonensis* 2506 isolate to MF *in vitro*

**Figure 1:**
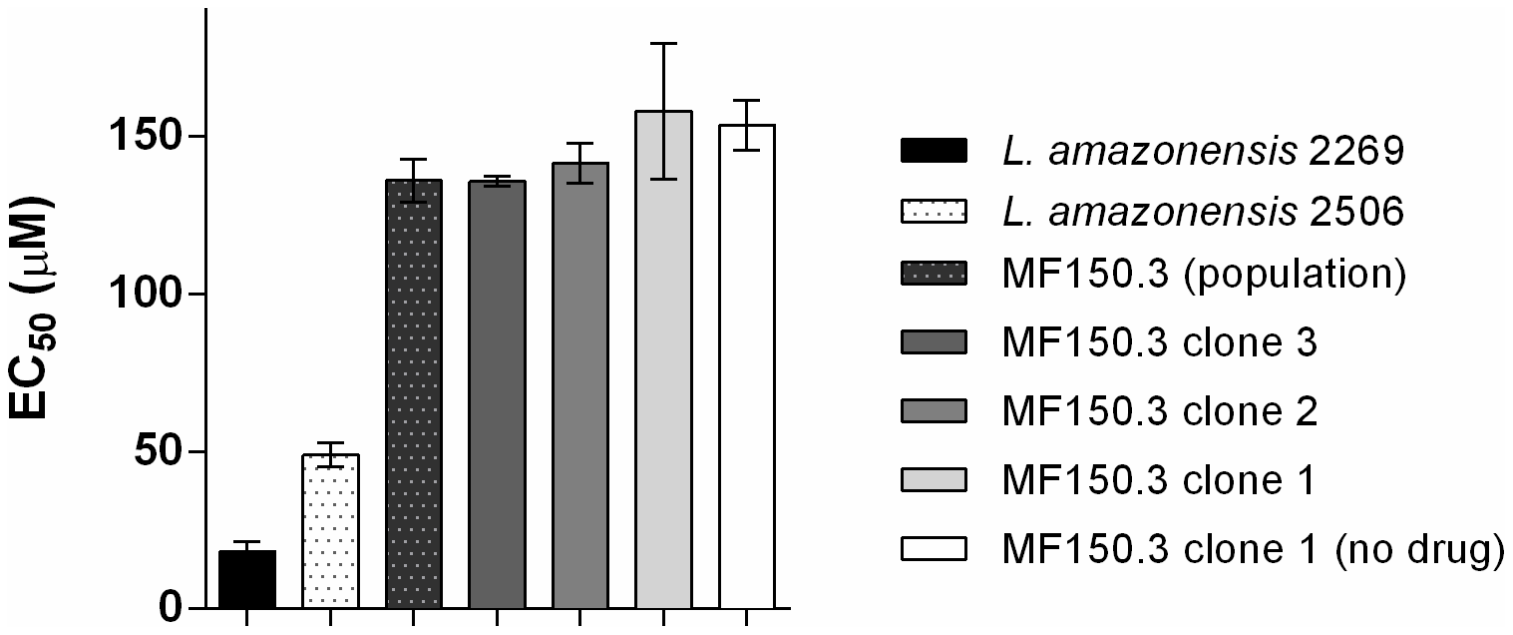
Activity of MF against promastigotes of *L. amazonensis*. MF susceptibility in promastigotes of *L. amazonensis* mutants and wild-type parasites. EC_50_ values for MF against M2269 strain, 2506 isolate and MF150.3-1 mutants were determined by MTT as described in Material and Methods. The average and standard deviation of three independent experiments in triplicate is shown.

The susceptibility tests on isolate 2506 were performed in parallel with a reference strain of *L. amazonensis* (M2269), isolated from a patient in the Amazon region (Pará state, Brazil) [17] and that has been maintained in culture for 40 years. Promastigotes of both strains had a similar pattern of growth *in vitro* (data not shown). The EC_50_ of MF against the 2506 isolate was found to be 2.4-fold higher than the type strain (Figure 1 and Table 1).

**Table 1:** Activity of MF against promastigotes and intracellular amastigotes of *L. amazonensis* wild-type strains and mutant MF150.3-1.

	Promastigotes		Amastigotes			
	Survival		% Infection^a^		Number of intracellular amastigotes^b^	
	EC_50_ c	EC_90_	EC_50_	EC_90_	EC_50_	EC_90_
***L. amazonensis*** ** M2269**	19.9±2.2	34.0	2.8±1.1	14.4	2.4±1.2	12.5
***L. amazonensis*** ** 2506**	47.0±3.9	64.1	12.7±4.1	23.5	10.6±4.2	32.8
**MF150.3-1**	167.3±16.8	204.3	>40	ND^d^	>40	ND

a EC_50_ determined according to the percentage of infection.

b EC_50_ determined according to the average number of amastigotes per infected macrophage.

c EC_50_ ± standard deviation in µM. Results are the average of at least three independent experiments.

d ND: not determined.

**Figure 2:**
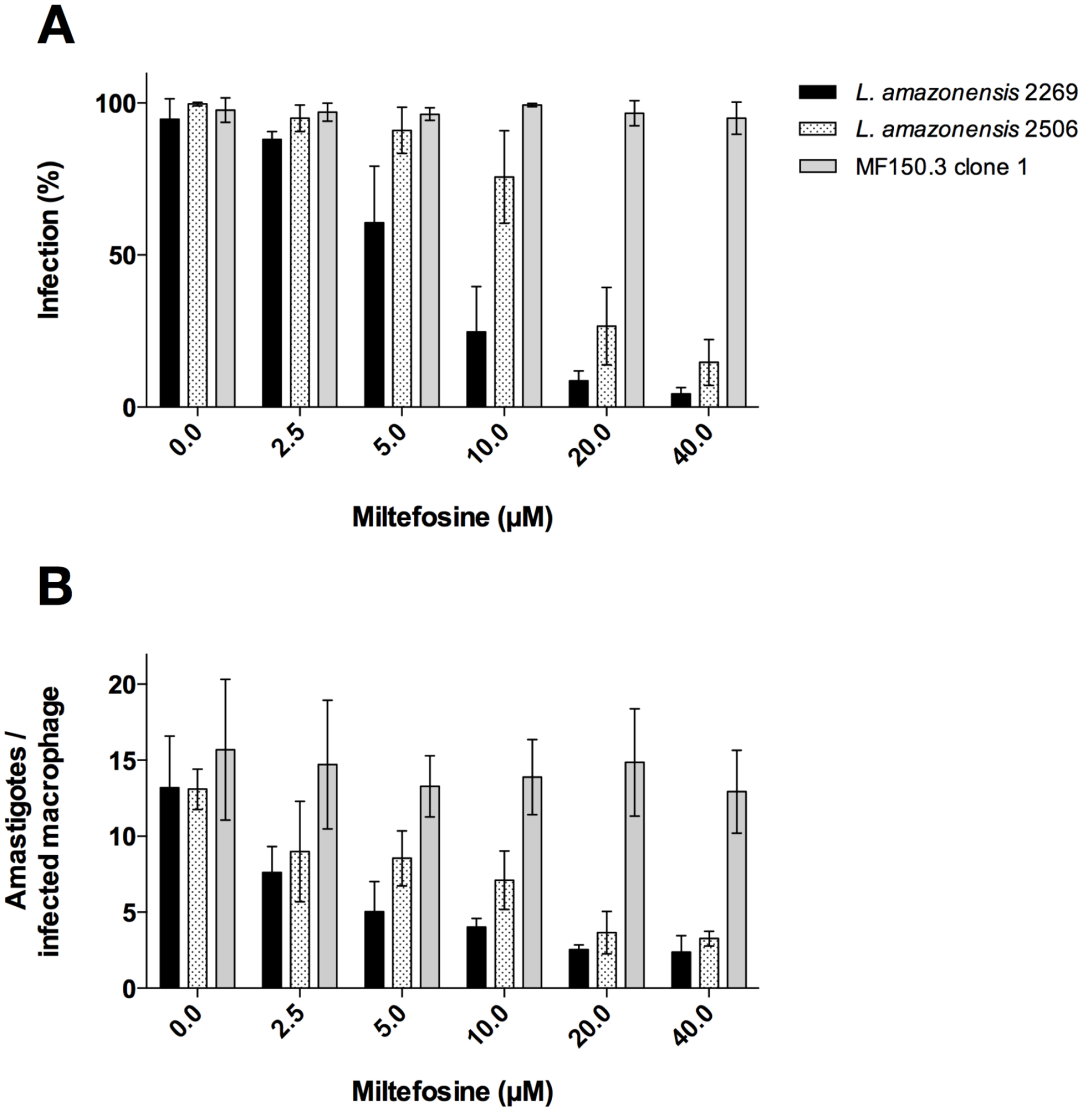
Activity of MF against intracellular amastigotes of *L. amazonensis*. (A) MF susceptibilitiy of intracellular amastigotes of *L. amazonensis* wild-type parasites M2269 and 2506 and mutant MF150.3-1. The percentage of infection was determined by counting 100 cells/coverslip and bars represent percentage of infected BMDM in different concentrations of MF as indicated. The average ± standard deviation of three independent experiments is shown. (B) Number of amastigotes per infected macrophage treated with different concentrations of MF (2,5–40 µM). Macrophages were infected with a ratio of 20 parasites per macrophage.

To verify whether the decreased sensitivity to MF observed for promastigotes of the isolate 2506 was also present in amastigotes, intracellular killing assays were performed comparing the reference strain M2269 with the isolate 2506. Initially, the percentage of infection and the average of amastigotes per infected BMDM were evaluated and compared between the two strains. *In vitro* infectivity to macrophages evaluated as the percentage of infected cells and number of parasites per infected cell were similar for the two lines (Figure S1). Treatment of infected BMDM with MF showed that the drug inhibited the *in vitro* intracellular growth of *L. amazonensis* M2269 and 2506 amastigotes in a dose dependent manner (Figure 2, A and B). The decreased susceptibility of the 2506 isolate, previously observed in promastigotes, was confirmed against intracellular amastigotes; *L. amazonensis* 2506 amastigotes were less sensitive to MF than the type strain, with a 4.5-fold increase in the EC_50_ (Table 1 and Figure 2A).

### *In vitro* selection of MF resistant *L. amazonensis*

The natural decreased susceptibility of the 2506 isolate to MF led us to ask whether previously described mechanisms of resistance to this drug would be used by *L. amazonensis*. To investigate that, we decided to characterize the natural 2506 isolate and to try and obtain *in vitro* selected drug resistant parasites, using a classical strategy for drug resistance selection. The mutant line MF150.3, selected using this strategy, exhibited an EC_50_ of MF 8.4-fold higher than the EC_50_ calculated for the reference wild-type strain (Figure 1 and data not shown). Three independent clones from this resistant population were obtained and tested for MF susceptibility. Similar levels of susceptibility were observed for the three clones (Figure 1), suggesting a homogenous resistance phenotype. We selected the MF150.3 clone 1 (MF150.3-1) to be further characterized. This mutant was highly resistant to MF compared to M2269 and 2506 strains (Table 1 and Figure 1) and this phenotype was stable, since parasites cultured in the absence of MF for 20 passages displayed the same level of resistance (Figure 1). As expected, amastigotes of the MF150.3-1 line were also highly resistant to MF (EC_50_ higher than 40 µM), with similar infection levels in untreated macrophages and in cells treated with the highest concentration of MF used in these experiments (Figure 2, A and B). It was not possible to determine the EC_50_ of this resistant line, because MF concentrations higher than 40 µM were toxic to macrophages (Figure 2A and data not shown).

### Molecular characterization of potential genes involved in MF susceptibility

To understand the basis for the altered susceptibility of the isolate and lines studied here, we chose to determine the nucleotide sequence of genes previously linked to MF resistance.

The sequences of *MT*, *Ros3* and *PK* encoding genes of M2269 strain, 2506 isolate and MF150.3 mutant were determined. A summary of these results is presented in Table 2.

**Table 2:** Nucleotide polymorphisms in *L. amazonensis* genes potentially involved in MF susceptibility/resistance in *Leishmania* a.

	*MT*	*Ros3*	*PK*
	(3,315 bp)^b^	(1,092 bp)^b^	(909 bp)^b^
*L. amazonensis* M2269	(108) GAG→E	-	**(614) CAC/CGC→H/R**
(reference strain)	(129) TAC/TAT→Y/Y		(678) TAT/TAC→Y
	(294) GGT/GGC→G/G		
	**(2555) GGG→G**		
	**(3271) CTT→L**		
*L. amazonensis* 2506	(108) GAG/GAA→E/E	No SNP	**(614) CAC→H**
(clinical isolate)	(294) GGC→G		(678) TAT→Y
	**(3271) ATT/CTT→I/L**		
MF150.3	(129) TAC→Y	No SNP	No SNP
(clones 1–3)	(294) GGC→G		
	**(2555) GAG→E**		

a SNPs found in the reference strain (*L. amazonensis* M2269) compared to the isolate *L. amazonensis* 2506 and to the MF150.3 resistant clones (1–3) are indicated. Non-synonymous SNPs are in bold.

b Gene size in base pairs (bp).

When the translated sequence of the 2506 MT was subjected to comparison against the M2269 sequence, 100% or 99.8% identity were detected depending on the allele examined. When compared to the ortholog in *L. mexicana*, sequences were 98.6% identical, while approximately 90% identity was obtained when comparing the *L. amazonensis* MT polypeptide sequence with the corresponding sequences from *L. major*, *L. infantum* or *L. donovani*.

Polymorphisms were found between *MT* and *PK* genes of the *L. amazonensis* 2506 isolate compared to the M2269 strain, with one non-synonymous SNP in each gene (Table 2). However, these amino acid changes were conservative and located outside the conserved motifs found in these proteins (Table 2). For *MT* and *PK* sequences, synonymous SNPs were also found while no SNP was found in the *Ros3* gene between these two parasites (Table 2). Therefore, no molecular signature was found in the genes previously associated with MF resistance that could justify the natural decreased susceptibility found in the 2506 isolate.

On the other hand, a single *MT* gene mutation at nucleotide position 2555 that causes an amino acid substitution, Gly-852→Glu (G852E) was present in three independent clones of the mutant line MF150.3 (Table 2 and data not shown). This amino acid substitution is located in the large cytosolic loop between the consensus sequences characteristics of P-type ATPases and the 5^th^ trans-membrane domain. Moreover, MF selection led to a loss of heterozigosity of the *MT* gene in MF150.3 clones, evidenced by the presence of just one allele (SNPs 129 and 294 in *L. amazonensis* M2269 reference strain represent independent alleles that became homozygous in the MF150.3 resistant strain) (Table 2). No SNPs were detected when nucleotide sequences of *Ros3* and *PK* genes were compared between the MF150.3 highly resistant mutant clones and the parental strain M2269 (Table 2).

### Analysis of phospholipid uptake

**Figure 3:**
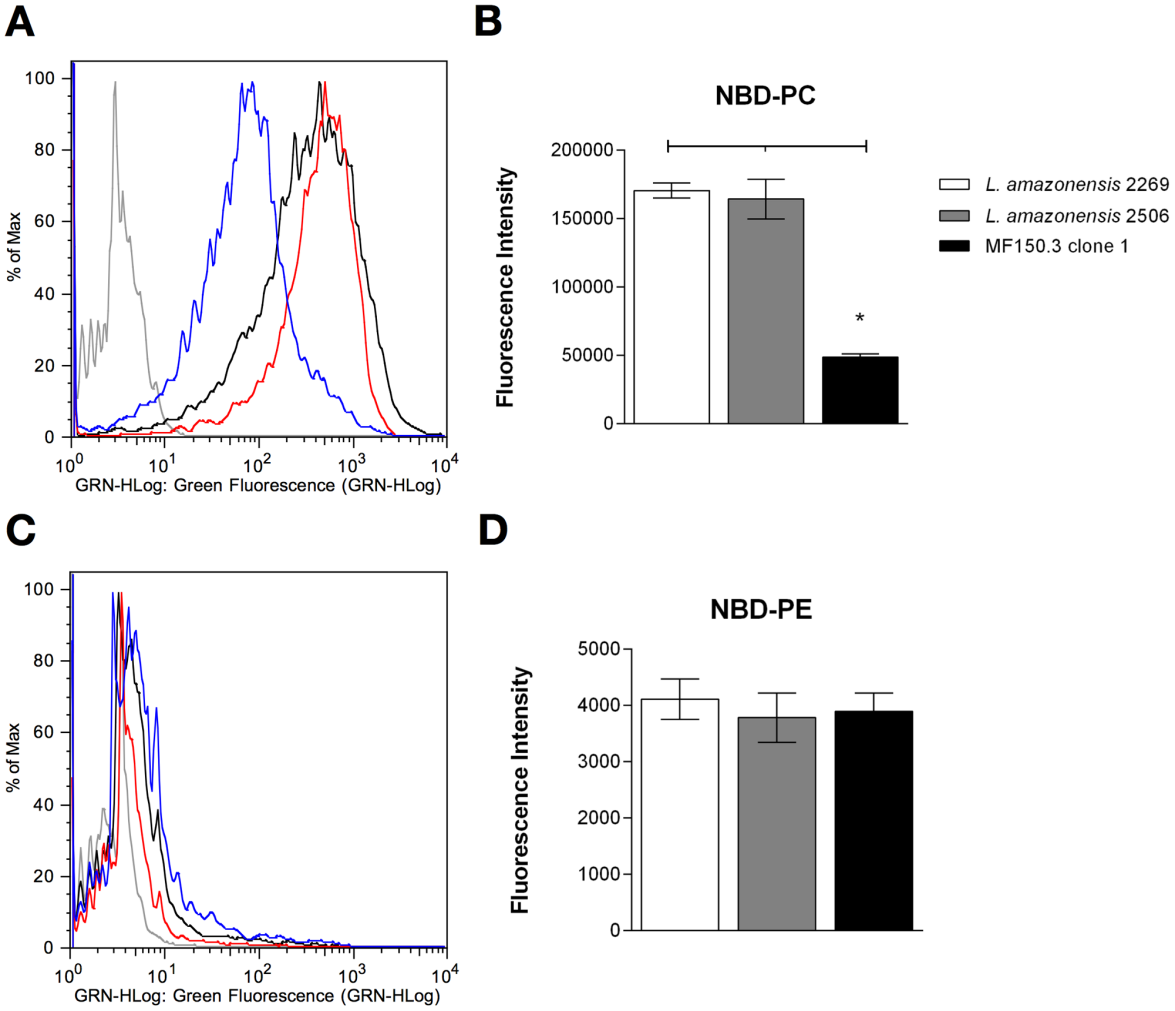
Phospholipid accumulation in *L. amazonensis* lines. Promastigotes were incubated with fluorescent phospholipid analogues NBD-PC or NBD-PE for 30 minutes at 25°C, washed and evaluated as described in the Methods by flow cytometry (A and C) or spectrofluorimetry (B and D). (A and C) Non-labelled parasites are shown in gray, while labelled parasites *L. amazonensis* M2269, 2506 and MF150.3-1 mutant with NBD-PC (A) or NBD-PE (C) are represented by black, red and blue lines, respectively. Histograms correspond to a representative experiment from three independent experiments. (B and D) Fluorescence intensity of labelled parasites with NBD-PC (B) or NBD-PE (D) measured by spectrofluorimetry. Unlabelled parasites were used as the blank reading. The average ± standard deviation of three independent experiments is shown. Statistical analysis was performed with One Way ANOVA, followed by the Tukey post-test. * p<0.01.

The MT is responsible for the translocation of phospholipids across the plasma membrane [10]. To verify whether the amino acid substitution identified in the *MT* gene of the mutant line could be correlated with an altered phenotype, we quantified the transport of fluorescent-labelled NBD-phospholipids in the reference strain, in the *in vitro* selected line and in the natural 2506 isolate. The accumulation of NBD-PC and NBD-PE was investigated by flow cytometry and fluorescence intensity. No significant differences in NBD-PC accumulation were found between M2269 and 2506 parasites (Figure 3, A and B). On the other hand, NBD-PC accumulation was approximately 5-fold lower in the MF150.3-1 line as compared with the parental strain M2269 (Figure 3, A and B). Therefore, a phenotypic change was apparent supporting a correlation between the reduced accumulation of PC and the G852E substitution in the *MT* gene in this MF-resistant line.

Strains M2269 and 2506 and MF 150.3-1 line displayed limited accumulation of NBD-PE (Figure 3, C and D). No significant differences were observed in the accumulation of PE among the three lines studied.

### *In vivo* efficacy of MF treatment

**Figure 4:**
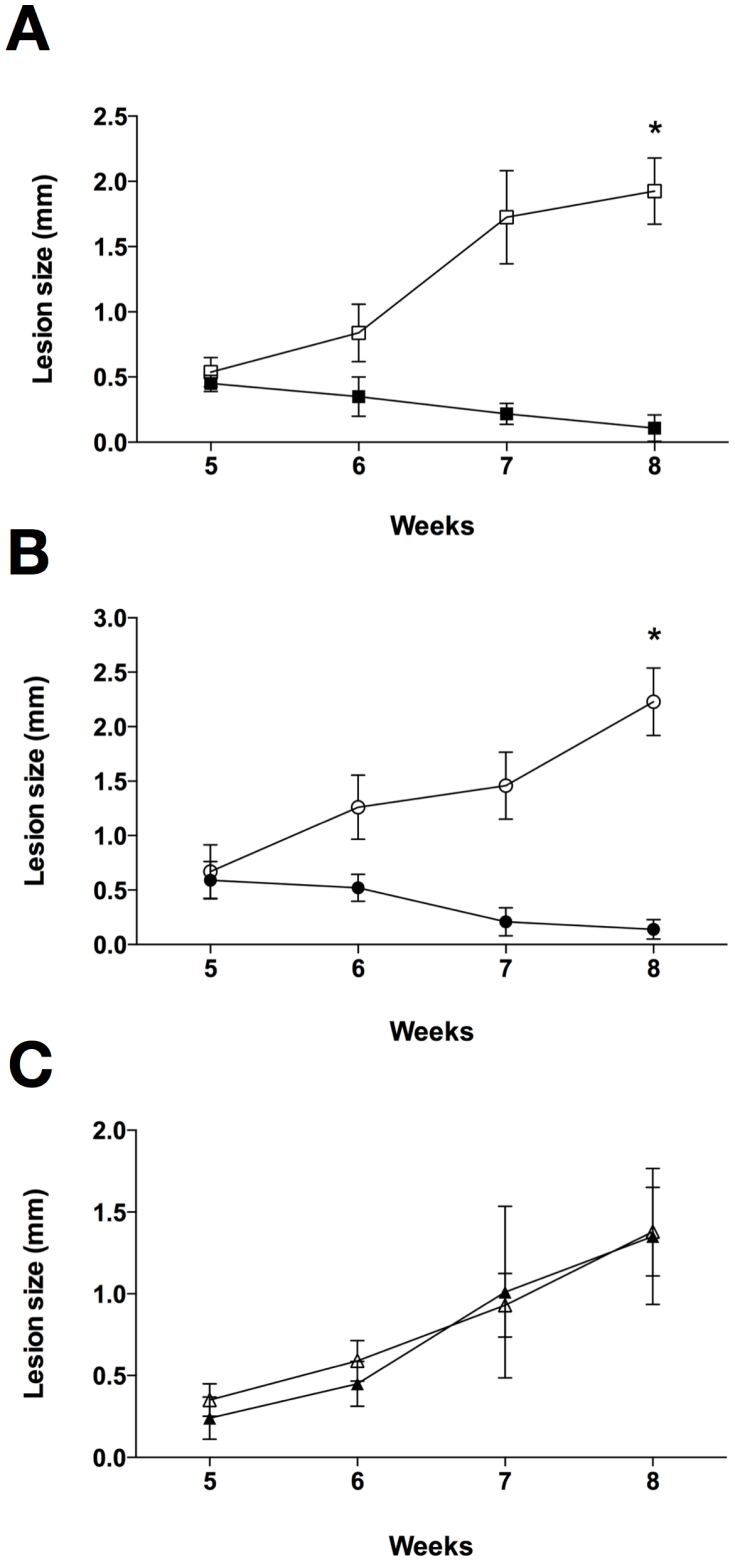
Evaluation of MF efficacy in mice infected with *L. amazonensis*. Lesion size represents the average difference between infected and contralateral non-infected hind footpads (five mice per group). Animals were treated with 13 mg/kg/day of MF for 15 consecutive days beginning five weeks post-infection. Animals infected with *L. amazonensis* M2269 strain (A), *L. amazonensis* 2506 isolate (B) and MF150.3-1 mutant (C) treated or not with MF (filled and open symbols respectively). Statistical analysis was performed with One Way ANOVA, followed by the Tukey post-test. * p<0.001.

**Figure 5:**
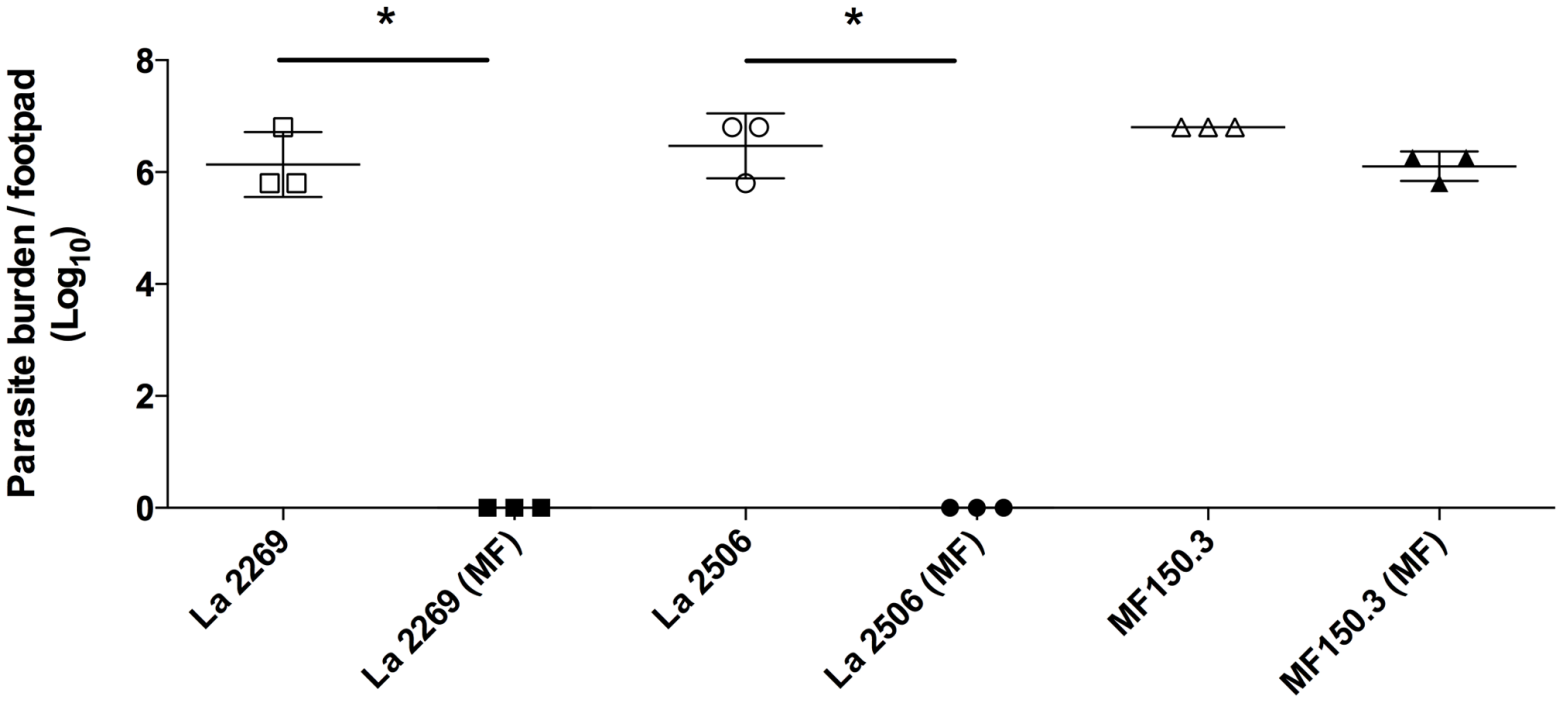
Effect of MF treatment on parasite burden in mice infected with *L. amazonensis* M2269, 2506 or the MF150.3-1 mutant line. Parasite burden was determined by limiting dilution at week 8 post-infection (one week after the end of MF treatment, for the MF-treated group) in 3 animals from each of the groups shown in Figure 4. Treated and untreated animals are represented by filled and open symbols respectively. Statistical analysis was performed with One Way ANOVA, followed by the Tukey post-test. * p<0.001.

To evaluate whether the decreased susceptibility observed *in vitro* for the isolate and for the mutant line would have an impact on the response to chemotherapy *in vivo*, we evaluated the efficacy of oral MF treatment in mice infected with the different parasites. When the control untreated groups were compared, no significant differences in the progression of disease were observed, as judged by the size of the lesion (Figure 4 and data not shown) and by the quantification of parasite burden in the three lines studied (Figure 5).

Infections with M2269 or 2506 parasites responded similarly to MF, with animals completely cured at the end of treatment (8 weeks post-infection) (Figure 4, A and B). Histopathological examination of tissues indicated that treatment of M2269 and 2506 infections with MF was translated into complete tissue healing with absence of detectable parasites (Figure S3). Limiting dilution assays confirmed the absence of parasites in the infected footpads of mice inoculated with M2269 or 2506 and treated with MF (Figure 5). No relapse was found in treated animals until 15 weeks after the interruption of treatment (data not shown).

On the other hand, infections with the MF150.3-1 line were completely refractory to MF treatment. MF150.3-1 infected mice treated with MF showed an indistinguishable progression of disease and parasite burden as compared with the control untreated group (Figures 4C, 5 and S3). These findings indicate that the resistant phenotype of MF150.3-1 line observed in promastigotes and intracellular amastigotes *in vitro* persisted *in vivo*.

To investigate whether the drug susceptibility parameters were stable after *in vivo* passage and maintenance in the absence of drug, parasites were recovered from mice infected with the MF150.3-1 line (treated with MF and untreated) 8 weeks post-infection and allowed to transform to promastigotes. The EC_50_ of MF for promastigotes recovered after infection from treated and untreated mice was 154.5±11.7 µM and 149.8±8.1 µM respectively (Figure S2A), therefore not significantly different from values determined prior to *in vivo* passage (Table 1). These parasites also maintained a reduced accumulation of NBD-PC as compared to the wild-type strain M2269 (Figure S2B). Finally, sequencing of the *MT* gene from the parasites isolated from treated and untreated mice confirmed the *MT* gene mutation at nucleotide position 2,555 (G852E substitution) (Figure S2C), previously identified in the selected resistant MF150.3-1 line used for infections in mice, confirming the stability of the mutation.

### Combined MF and pentamidine in the treatment of a patient with DCL

Based on renal failure grading, susceptibility assays and drug availability, a course of treatment was administered to the DCL patient associating MF and pentamidine. Miltefosine 100 mg bid was given for 40 days while 10 doses of 200 mg pentamidine (3 mg/kg/day) were administered over 60 days. The patient responded with a sharp decrease in ulcerated areas.

## Discussion

This work was driven by the necessity of finding a new therapeutic scheme for a DCL patient that, over 25 years, had received multiple courses of antimony and amphotericin B, with only partial responses to chemotherapy. The therapeutic options for the treatment of this patient were very limited. Antimonials were ineffective and renal insufficiency precluded further use of amphotericin B. MF was then contemplated as a possible alternative.

The initial evaluation of the clinical isolate indicated, however, that *L. amazonensis* 2506 was less suscetible to MF when compared with the reference strain *in vitro*. The EC_50_ were 2.4 and 4.5-fold greater in 2506 promastigotes and amastigotes, respectively, as compared with the M2269 type strain. These findings confirm previous observations reporting variable MF sensitivity in *Leishmania* species and strains [28]–[31]. It should be emphasized that the 2506 strain was never exposed to MF, indicating that these parasites are intrinsically more tolerant to MF.

We then decided to compare the phenotype of this less susceptible isolate with parasites selected for MF resistance *in vitro*. Stepwise increase in MF concentrations led to the selection of promastigotes displaying 8.4-fold increased EC_50_s in about 30 passages.

Previous studies have demonstrated that the inactivation of MT through point mutations is a trait found in *L. donovani*, *L. major* and *L. infantum* resistant mutants selected *in vitro* [10], [11]. Similarly, in the *L. amazonensis* resistant mutant MF150.3-1 a single point mutation in the *MT* gene was identified, leading to an amino acid substitution (G852E). Both alleles were mutated owing to a loss of heterozigosity observed in all clones isolated from the initial MF150.3 population, suggesting the initial selection of a homogenous population (data not shown). This amino acid change is located in the large cytosolic loop between the consensus sequences characteristic for P-type ATPases and the 5^th^ transmembrane domain. This particular region of the protein seems important for MF transport, since other *Leishmania* MF resistant mutants contained mutations in the same region. In *L. donovani* and *L. major* selected resistant mutants, respectively L856P and G852D substitutions in *MT* sequence were found [10], [11]. Interestingly, a single point mutation in the same region (L832F) was also identified in *L. infantum* isolated from an HIV co-infected patient after treatment with MF [32]. On the other hand, we did not find evidence of a MT inactivation on the 2506 isolate.

Other genes potentially involved in MF resistance were also sequenced in this study. These genes were selected based on recent findings using whole genome sequencing technology in *L. major* MF resistant mutants selected *in vitro* [11]. We looked for mutations/polymorphisms in *Ros3* and *PK* genes in *L. amazonensis* 2506 isolate and MF150.3 line. No SNP was found in the *Ros3* gene in the parasites studied.

PK is responsible for the phosphorylation of pyridoxal 5′phosphate, the active form of vitamin B6 [33]. The *PK* gene was found mutated in *L. major*, but not in *L. infantum* lines resistant to MF [11]. In this study, no mutation in the *PK* gene was found in three independent clones of the MF150.3 line. This gene was in heterozygosity in the type strain M2269 and contained two SNPs, while in 2506 strain just one allele was present with these SNPs in homozygosity. One of these SNPs codes for two different amino acids, however this change is conservative and not located in the conserved motifs of PK.

In agreement with the MT sequence data, the accumulation of NBD-PC was reduced in the MF150.3 line but not in the 2506 isolate, as compared with the reference strain M2269. A reduced accumulation of PC was previously described in *L. donovani* MF resistant mutants displaying *MT* gene inactivation [10]. On the other hand, these same *L. donovani* resistant mutants had a reduced accumulation of PE [10]. In *L. amazonensis* the accumulation of NBD-PE was extremely low and did not change when the reference strain was compared with the parasites less susceptible to MF. Differences in phospholipid internalization patterns have been noted for different *Leishmania* species [13] and may be due to the MT activity and substrate specificity.

These analyses indicated, therefore, that while in *L. amazonensis* mutations in the *MT* are related to MF resistance as shown previously for other species, this was not the cause of the reduced MF tolerance of the 2506 isolate.

Recently, an alternative method was described to obtain resistant parasites using intracellular amastigotes *in vitro* [34], [35]. This method mimics the disease more closely and was employed to select paromomycin resistant parasites [35]. Interestingly, when amastigotes were selected by exposure to MF, no shift in the EC_50_ was found. However, promastigotes differentiated from these selected amastigotes were harvested at increased MF concentrations as compared to the control parasites [34]. In our study, the decreased susceptibility to MF was a stable phenotype observed equally for promastigotes and amastigotes. The different resistant patterns observed in parasites selected using different methods support the existence of more than one mechanism leading to MF resistance.

Notwithstanding the success in selecting *L. amazonensis* resistant mutants *in vitro*, we were still left with a problem. Our data showed that *L. amazonensis* 2506 was less susceptible to MF than the M2269 type strain and that this phenotype was not due to mutations in the *MT* gene or in other genes previously linked to MF resistance. What was the significance of the decreased susceptibility? Was this resistance or, in other words, was this level of reduction in susceptibility sufficient to impair the clinical outcome if MF was used?

The outcome of treatment was then compared in BALB/c mice infected with the *L. amazonensis* type strain, with the 2506 isolate or with the MF150.1 line. The World Health Organization recommends 2.5 mg/kg/day MF for 28 days for the treatment of CL or VL in humans. This scheme is not effective for obtaining complete cure in *L. amazonensis* infected BALB/c mice [36]. Previous studies in our laboratory indicated that MF was effective in the treatment of *L. amazonensis* infections in BALB/c mice if used at 13 mg/kg/day for 15 days by the oral route (data not shown).

After MF treatment, we observed clinical and parasitological cure in mice infected with *L. amazonensis* M2269 and 2506. On the other hand, infections with MF150.3-1 were completely refractory to the same MF dose, with similar number of parasites recovered at the end of treatment in treated and untreated animals. Moreover, promastigotes recovered from lesions of treated and untreated MF150.3-1 infected animals were equally resistant to MF. Similar findings were described in a *L. donovani* resistant line that contained the *MT* gene mutated, with no response to MF therapy in mice [37]. Based on these findings, we can state that MT has an essential role in MF susceptibility *in vivo* across the genus *Leishmania* and its activity may affect MF response *in vivo*.

Previous reports studying the correlation between *in vitro* and *in vivo* suscetibility/outcome of therapy in leishmaniasis showed no correlation between *in vitro* susceptibility to antimonials and the corresponding *in vivo* treatment outcome [38], [39]. Similar findings were described here. Promastigotes and amastigotes of the 2506 isolate were less susceptible to MF when compared with the reference strain M2269 *in vitro* but not *in vivo*, prompting the reevaluation of MF's prospects in the treatment of the DCL patient. The administration of an association of MF and pentamidine was employed successfully, leading to healing of most of the cutaneous lesions (data not shown). Obviously, long-term efficacy is still uncertain and will have to be evaluated during the clinical follow-up in months to come.

The characterization of clinical or field isolates of *Leishmania* always raises concerns related to the potential changes in phenotype determined by the adaptation to laboratory conditions. Even when restricting the analysis to parasites with a low passage *in vitro*, we cannot completely rule out differences between parasites inhabiting the natural hosts and the ones obtained from culture. As far as the parameters evaluated in this study were concerned, the isolate 2506 maintained the growth pattern, infectivity and virulence observed previously for other clinical isolates [24].

In conclusion, our findings showed that MF was effective *in vivo* against a *L. amazonensis* isolate obtained from a Brazilian DCL patient, despite the decreased pattern of MF susceptibility *in vitro*. The mechanisms that justify the 2506 isolate decreased susceptibility to MF *in vitro* are still undetermined. On the other hand, MT activity proved to be essential for drug effectiveness, with parasites becoming completely refractory when this transporter was inactivated. These findings highlight the need to consider *in vitro* resistance determinations with caution as well as the relevance of MT as a potential molecular marker of MF unresponsiveness in leishmaniasis patients.

## Supporting Information

Figure S1 *In vitro* infectivity of *L. amazonensis* M2269, 2506 and MF150.3-1. BMDM were infected with stationary phase promastigotes for 3 h at 33°C. Non-internalized parasites were removed by washing with warmed PBS. After 72 h, cells in 24-well chamber slides were fixed in methanol, stained and infected macrophages and amastigotes were determined by counting 100 cells. The average ± standard deviation of three independent experiments is shown. (A) Percentage of infected macrophages in infections initiated with a ratio of 5, 10 or 20 parasites/macrophage. (B) Number of amastigotes per infected macrophage in infections as in (A).
(TIF)

Figure S2 Characterization of MF150.3-1 lines isolated from infected mice treated or not with MF [MF150.3 clone 1 (MF) and MF150.3 clone 1 (NT) respectively]. MF150.3-1 lines were isolated from infected footpads 8 weeks post-infection. Amastigotes were differentiated in M199 medium to promastigotes and experiments were performed during the first 5 passages *in vitro*. (A) MF susceptibility of MF150.3-1 (NT) and MF150.3-1 (MF) lines. The average ± standard deviation of three independent experiments in triplicate is shown. (B) Flow cytometry analysis of NBD-PC accumulation in MF150.3-1 lines. Non-labelled parasites are shown in gray, while labelled parasites, *L. amazonensis* 2269, MF150.3-1 (NT) and MF150.3-1 (MF) are represented in black, red and blue respectively. Histogram corresponds to a representative experiment from three independent experiments. (C) Partial nucleotide sequence alignment of *MT* genes of *L. amazonensis* 2506 and 2269 strains and the mutant MF150.3-1 that contains a point mutation at position 2,555 (nucleotide in red). Nucleotide sequences of the *MT* gene of MF150.3-1 (NT) and MF150.3-1 (MF) lines are also indicated in the alignment. Nucleotide sequences correspond to consensus sequences of at least three independent sequencing reactions at that particular region.
(TIF)

Figure S3 Histological analysis of the infection sites in mice inoculated with *L. amazonensis* M2269, 2506 or MF150.3-1 mutant after treatment or untreated with MF (8 weeks post-infection). Infected footpad fragments were washed with PBS, fixed with formalin and processed with paraffin. Sections were stained with haematoxylin-eosin and then visualized in a light microscope. *L. amazonensis* M2269 strain treated (A) and untreated with MF (B–C); *L. amazonensis* 2506 isolate treated (D) and untreated with MF (E–F) and MF150.3-1 mutant untreated (G–I) and treated with MF (J–L). Bars: 50 µm (A, D, G and J), 20 µm (B, E, H and K) and 10 µm (C, F, I and L).
(TIF)

Table S1 Primers used for amplifying and sequencing the genes *MT*, *PK* and *Ros3* of *L. amazonensis* lines.
(PDF)
