# Supplementary figures and images for: In Vitro and In Vivo Miltefosine Susceptibility of a Leishmania amazonensis Isolate from a Patient with Diffuse Cutaneous Leishmaniasis
Source: PLoS Negl Trop Dis. 2014 Jul 17;8(7):e2999. doi: 10.1371/journal.pntd.0002999 (PMC4102453; doi:10.1371/journal.pntd.0002999)

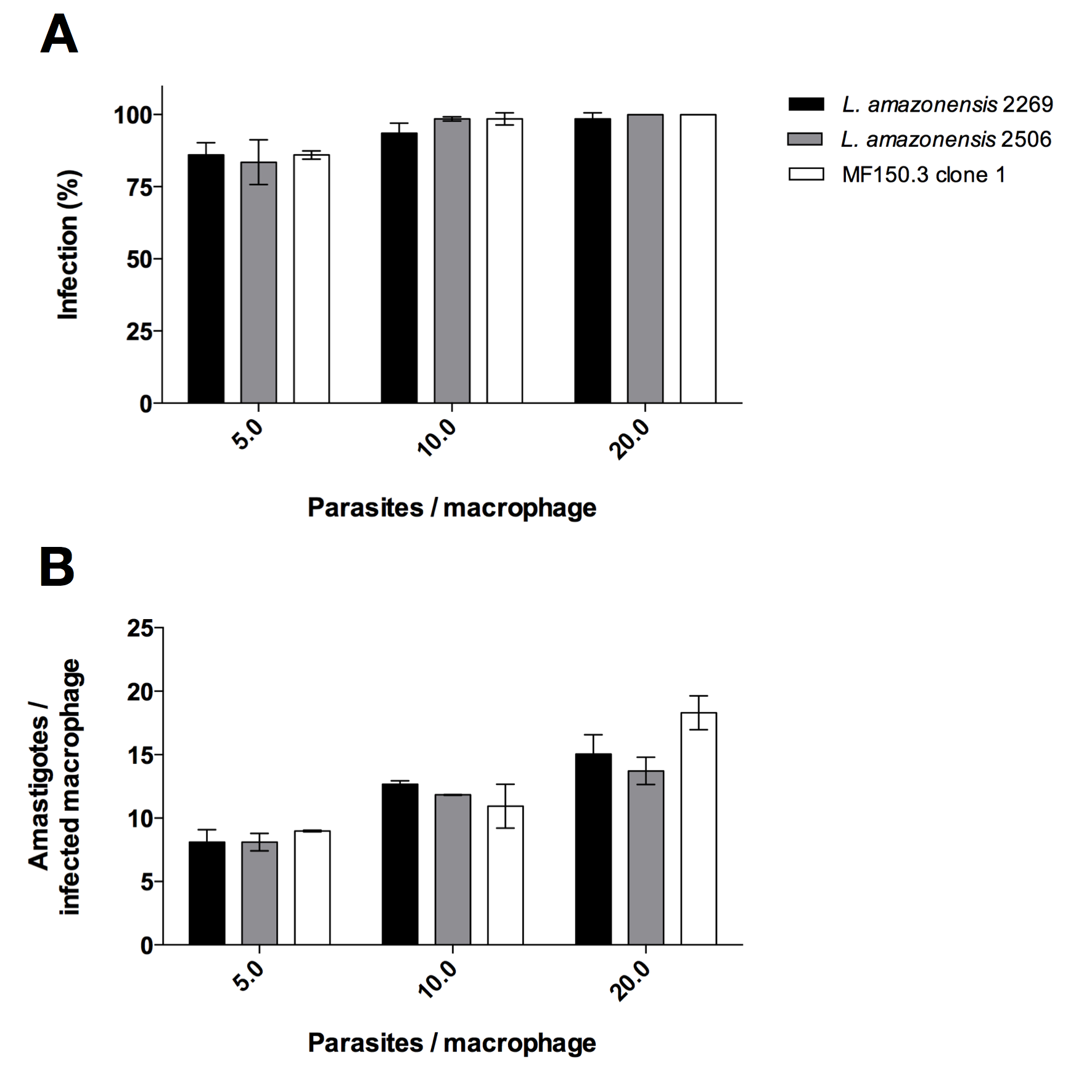

Supplement: Figure S1 — In vitro infectivity of L. amazonensis M2269, 2506 and MF150.3-1. BMDM were infected with stationary phase promastigotes for 3 h at 33°C. Non-internalized parasites were removed by washing with warmed PBS. After 72 h, cells in 24-well chamber slides were fixed in methanol, stained and infected macrophages and amastigotes were determined by counting 100 cells. The average ± standard deviation of three independent experiments is shown. (A) Percentage of infected macrophages in infections initiated with a ratio of 5, 10 or 20 parasites/macrophage. (B) Number of amastigotes per infected macrophage in infections as in (A). (TIF) [file pntd.0002999.s001.tif]

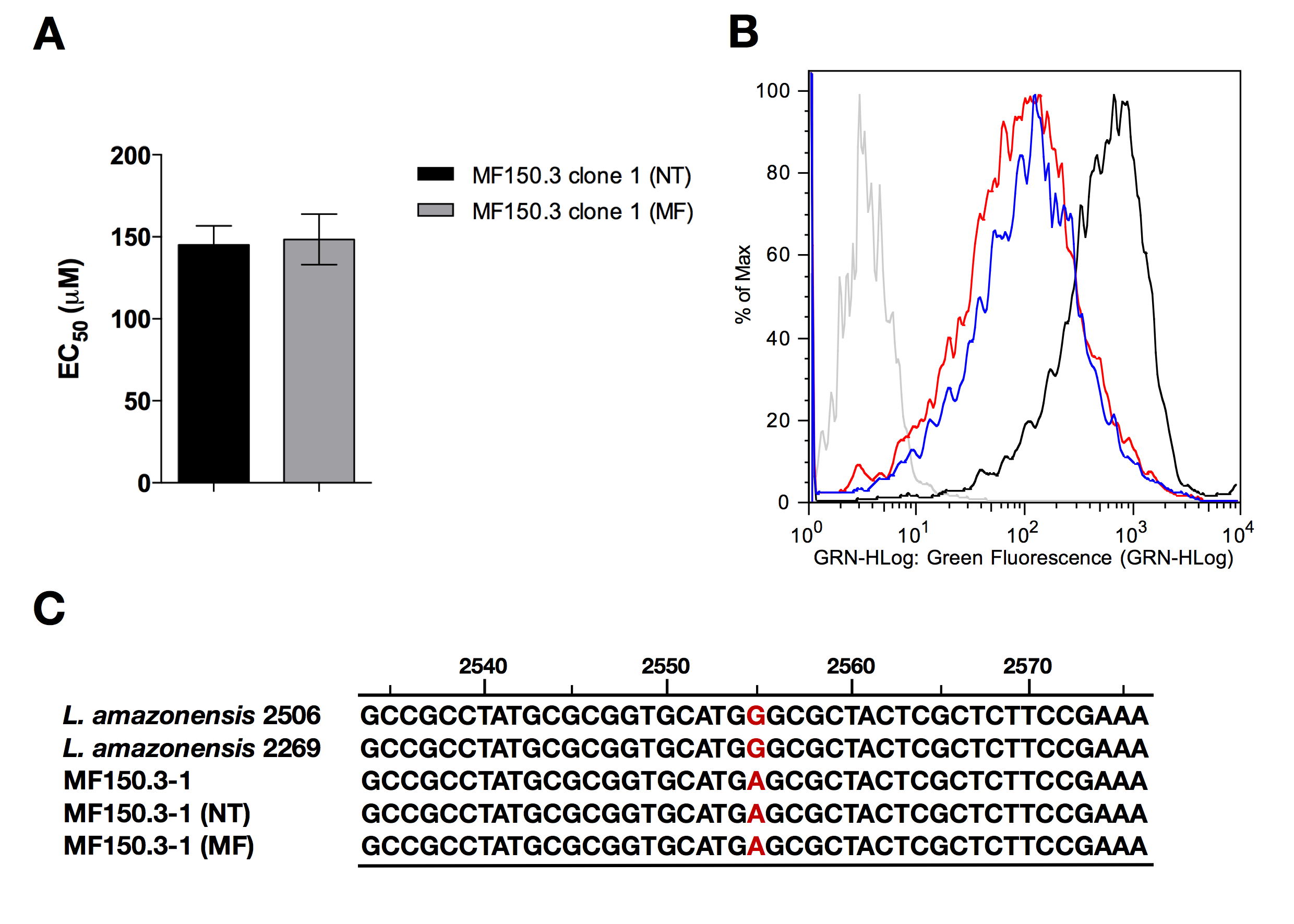

Supplement: Figure S2 — Characterization of MF150.3-1 lines isolated from infected mice treated or not with MF [MF150.3 clone 1 (MF) and MF150.3 clone 1 (NT) respectively]. MF150.3-1 lines were isolated from infected footpads 8 weeks post-infection. Amastigotes were differentiated in M199 medium to promastigotes and experiments were performed during the first 5 passages in vitro. (A) MF susceptibility of MF150.3-1 (NT) and MF150.3-1 (MF) lines. The average ± standard deviation of three independent experiments in triplicate is shown. (B) Flow cytometry analysis of NBD-PC accumulation in MF150.3-1 lines. Non-labelled parasites are shown in gray, while labelled parasites, L. amazonensis 2269, MF150.3-1 (NT) and MF150.3-1 (MF) are represented in black, red and blue respectively. Histogram corresponds to a representative experiment from three independent experiments. (C) Partial nucleotide sequence alignment of MT genes of L. amazonensis 2506 and 2269 strains and the mutant MF150.3-1 that contains a point mutation at position 2,555 (nucleotide in red). Nucleotide sequences of the MT gene of MF150.3-1 (NT) and MF150.3-1 (MF) lines are also indicated in the alignment. Nucleotide sequences correspond to consensus sequences of at least three independent sequencing reactions at that particular region. (TIF) [file pntd.0002999.s002.tif]

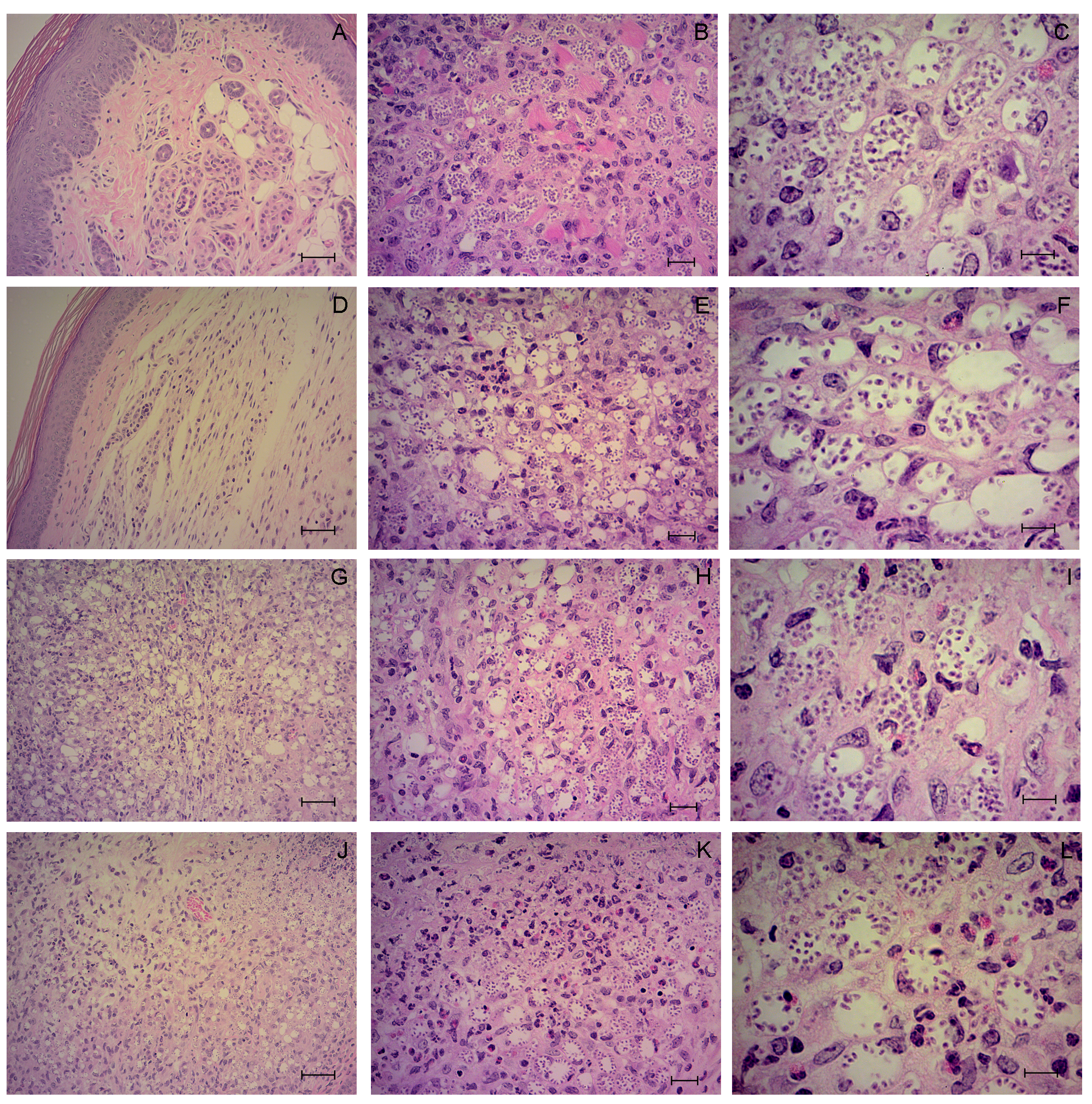

Supplement: Figure S3 — Histological analysis of the infection sites in mice inoculated with L. amazonensis M2269, 2506 or MF150.3-1 mutant after treatment or untreated with MF (8 weeks post-infection). Infected footpad fragments were washed with PBS, fixed with formalin and processed with paraffin. Sections were stained with haematoxylin-eosin and then visualized in a light microscope. L. amazonensis M2269 strain treated (A) and untreated with MF (B–C); L. amazonensis 2506 isolate treated (D) and untreated with MF (E–F) and MF150.3-1 mutant untreated (G–I) and treated with MF (J–L). Bars: 50 µm (A, D, G and J), 20 µm (B, E, H and K) and 10 µm (C, F, I and L). (TIF) [file pntd.0002999.s003.tif]
