# Supplementary material for: In Vitro and In Vivo Miltefosine Susceptibility of a Leishmania amazonensis Isolate from a Patient with Diffuse Cutaneous Leishmaniasis
Source: PLoS Negl Trop Dis. 2014 Jul 17;8(7):e2999. doi: 10.1371/journal.pntd.0002999 (PMC4102453; doi:10.1371/journal.pntd.0002999)
Supplement: Table S1 — Primers used for amplifying and sequencing the genes MT, PK and Ros3 of L. amazonensis lines. (PDF) [file pntd.0002999.s004.pdf]

Table S1. Primers used in this study for amplifying and sequencing *L. amazonensis MT, PK* and *Ros3* genes. Primers were designed based on *L. mexicana* (MHOM/GT/2001/U1103) nucleotide sequences available at TriTrypDB (http://www.tritrypdb.org/). *L. mexicana* gene ID for *MT, PK and Ros3* are LmxM.13.1530, LmxM.29.1250 and LmxM.31.0510, respectively. Restriction sites are shown in bold.

LmxM.13.1530 F (*Xba*I)	5' CGC**TCTAGA**CACCACCACCACTCCTGCCT
LmxM.13.1530 R (*Hind*III)	5' CGC**AAGCTT**TCTGCTCACGTTCCGCCCTC
LmxM.13.1530 F1 (internal - position 736)	5' GAGGAGCACGCACTCTCGCT
LmxM.13.1530 F2 (internal - position 1432)	5' CAGCCCTTCAAGGACGACAC
LmxM.13.1530 F3 (internal - position 2097)	5' GCGCAACGACTTCATCGACC
LmxM.13.1530 R1 (internal - position 2491)	5' CGGCATGTGCACCTTCCAGC
LmxM.13.1530 R2 (internal - position 1942)	5’ ATGGCGGTGGCGCCGACGAG
LmxM.13.1530 R3 (internal - position 1106)	5’ GTGACGAACAGCGAGATGGG
LmxM.29.1250 F	5’ CGGAAAGGGGCGAATCCATTGGT
LmxM.29.1250 R	5’ CTGAAAAGGGCGCCAACCCC
LmxM.31.0510 F	5’ TCGTGGGCCAAATCATGGCGT
LmxM.31.0510 R	5’ TGCATTTTGGCTTCACGAGAAAGGCG
